# Early experience with a novel transapical transcatheter aortic valve system in patients with severe aortic stenosis: a prospective, multicenter study

**DOI:** 10.3389/fcvm.2025.1457180

**Published:** 2025-03-21

**Authors:** Lulu Liu, Jian Yang, Haibo Zhang, Jian Liu, Yucheng Zhong, Lai Wei, Xianbao Liu, Zhao Jian, Yuan Zhao, Yingqiang Guo

**Affiliations:** ^1^ Department of Cardiovascular Surgery, West China Hospital, Chengdu, China; ^2^ Department of Cardiac Surgery, Xijing Hospital, Xi'an, China; ^3^ Department of Cardiac Surgery, Beijing Anzhen Hospital, Beijing, China; ^4^ Department of Cardiac Surgery, Guangdong Provincial People’s Hospital, Guangzhou, China; ^5^ Department of Cardiovascular Medicine, Wuhan Union Hospital, Wuhan, China; ^6^ Department of Cardiac Surgery, Shanghai Zhongshan Hospital, Shanghai, China; ^7^ Department of Cardiovascular Medicine, The Second Affiliated Hospital of Zhejiang University, Hangzhou, China; ^8^ Department of Cardiac Surgery, Xinqiao Hospital, Chongqing, China; ^9^ Department of Cardiac Surgery, The Second Xiangya Hospital of Central South University, Changsha, China

**Keywords:** aortic stenosis, transcatheter aortic valve replacement, transapical, Xcor transcatheter heart valve system, supporting arm

## Abstract

**Objectives:**

Registered, prospective, multicenter study of the short-term clinical outcomes of a novel transcatheter aortic valve system (Xcor system, Saint Medical Technology, Inc., Nanjing) to evaluate its safety and efficacy.

**Methods:**

130 high risk patients with symptomatic severe AS from 11 institutions were treated with the novel Xcor system. All patients were pre-TAVR assessed by transthoracic echocardiography and computed tomography of the aortic valve (AV) and relevant left cardiac and vascular anatomy. Procedural, in-hospital, and follow-up clinical outcomes were evaluated after procedures.

**Results:**

The average age of the 130 patients was 71.2 ± 4.4 years old, 55.4% were male, and the STS score was 8.0 ± 3.9%. Device and procedural success were achieved in 98.5% and 97.7% of the patients, respectively. At 30-day follow-up, all-cause mortality, the incidence of major adverse cardiovascular events, major vascular complications, and new permanent pacemaker implantation were 3.8%, 4.6%, 0.8%, and 0.8%, respectively. 7.7% of patients showed ≥ mild paravalvular leakage, and all 125 (100%) patients were in New York Heart Association Class ≤ II. The procedural and clinical outcomes of bicuspid AV patients were similar to those of tricuspid AV patients.

**Conclusions:**

Overall, the 30-day follow-up shows that the procedural outcomes with the novel Xcor system with self-centering support arms are comparable or superior to other contemporary TAVI devices, with a low all-cause mortality, low major adverse cardiovascular events, low PVL and similar clinical outcomes for BAV and TAV patients.

## Introduction

Transcatheter aortic valve replacement (TAVR), as an alternative treatment to surgical aortic valve replacement (SAVR) for severe aortic stenosis (AS), has been proven to be effective and safe (1). With the continuous expansion of clinical indications (2, 3), TAVR has recently become a treatment option for patients with severe AS at high, intermediate and low risk for surgery (1–5).

However, existing transcatheter heart valve systems (THV) rely primarily on the radial force generated between the device and the aortic root. Due to the complex anatomical structures of the aortic root in AS patients, radial force alone may not prevent displacement or malpositioning, which may seriously affect the procedural outcomes and prognosis (6, 7). The Xcor system (Saint Medical Technology Co., LTD., Nanjing, China) with its innovative design of supporting arms may enable improved control of THV in the optimal position to achieve form-fitting to the aortic root.

The aim of this study was to introduce the novel Xcor system and evaluate the efficacy and safety of this transapical, transcatheter system in 130 patients with severe AS in a premarket, registered, prospective, multicenter trial.

## Methods

### Study population

A total of 130 patients met the inclusion criteria for the study at 11 institutions in China between May 2022 to May 2023. All patients were evaluated by specialized cardiac teams at each center prior to admission and were considered prohibited or high-risk for surgical aortic valve replacement (SAVR). The study protocol was approved by the Ethics Committee of West China Hospital (HX-2022-17) and other institutional ethics committees at all participating sites. Inclusion criteria included: (1) Age ≥ 70 years old; (2) Severe AS: Mean pressure gradient (PG) ≥ 40 mmHg (1 mmHg = 0.133 kPa) or aortic annular area <1.0 cm^2^; (3) New York Heart Association Class (NYHA) ≥ II; 4) High risk for surgical aortic valve replacement. Exclusion criteria included: (1) Acute myocardial infarction occurred within 1 month; (2) Aortic root anatomy and pathological changes were not suitable for bioprosthetic valve implantation; (3) Any therapeutic traumatic heart surgery within 1 month; (4) Ascending aortic aneurysm diameter ≥50 mm; (5) Left ventricular ejection fraction (LVEF) < 20%; (6) Echocardiography indicated the presence of intracardiac mass, thrombus, or neoplasm; (7) Infectious endocarditis, etc. Detailed criteria are listed in Supplementary Table S1.

Transthoracic echocardiography (TTE) and computed tomography (CT) were conducted prior to the procedures. CT assessments included the morphology of the aortic valve (AV) and the aortic root. The angle between the major axis of the aortic root and the left ventricle outflow tract (LVOT) was measured to determine the optimal angle for the delivery system. For patients with tricuspid AV (TAV), the implanted Xcor size was selected based on the annular measurements. For patients with bicuspid AV (BAV) or severe calcification, the implanted Xcor size was selected based on the super-annular measurements. Additionally, the distances between the annulus and the left/right coronary orifices were measured. TTE measured the mean pressure gradient to classify the severity of AS.

### Study device

The Xcor transcapical system uses a self-expanding nitinol frame designed in a monolithic fashion with 6 expandable arms symmetrically arranged in the middle part of the stent. At the time of study the system is available in 3 different sizes (23, 26 and 29 (measured as the maximum diameter of the inflow) covering an annular range between 19 and 28 mm (Supplementary Figure S1).

**Figure 1:**
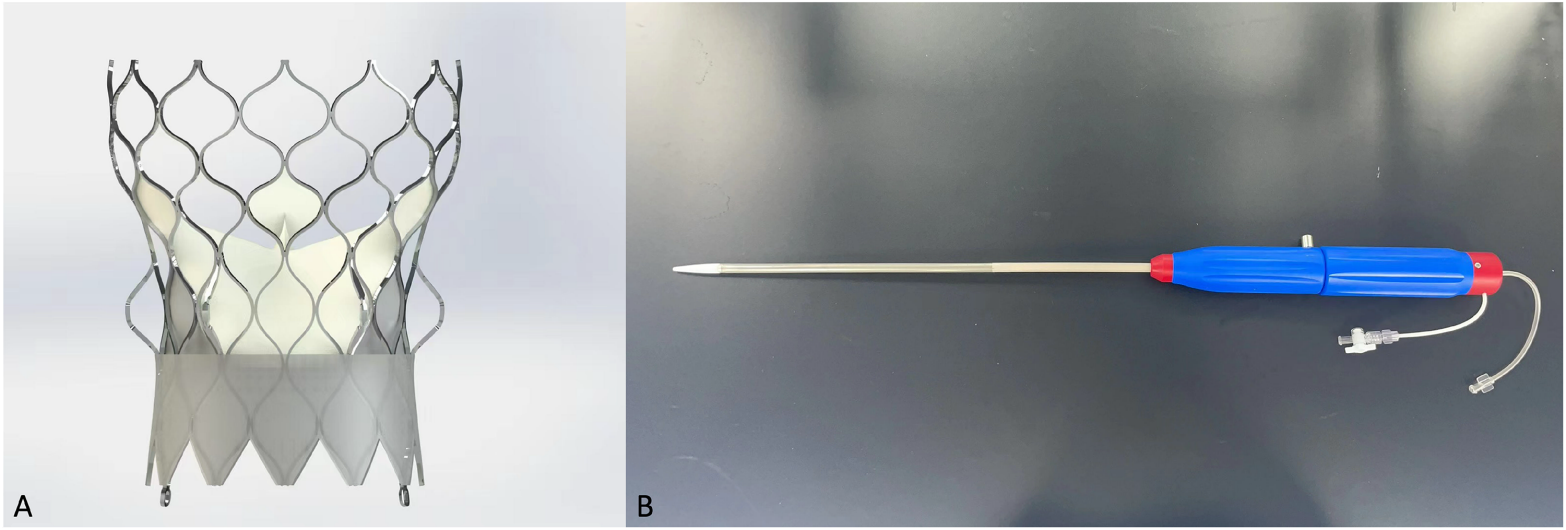
Transcatheter heart valve design. Xcor. **(A)** The Xcor prosthesis is composed of a self-expanding nitinol stent, three pieces of bovine leaflets, and the polymer skirt. The yellow circles represent the uniquely designed supporting arms. **(B)** The delivery system.

Key aspect of the Xcor stents are the above mentioned expandable arms. Designed with no undercut, allowing therefore for full recoverability, their role is to assure the correct positioning, self -alignment and anchoring of the prosthesis (Figures 1A,B). Using a form-fitting principal the arms allow to reduce the maximum radial force of the conical inflow of the stent. In combination with assuring a minimum protrusion inside the LVOT, these two aspects avoid any pressure on the LBB.

Assuring a supra-annular valve function of the three glutaraldehyde-treat bovine pericardial leaflets the expendable arms in conjunction with large cells preserve and facilitate coronary access. Central co-axial self-alignment and the Dacron outer-skirt with a total height of 10–11 mm confer an excellent seal preventing paravalvular leakage. All valve sizes can be used with the same 23 French delivery system. Designed in a minimalist and straight-forward fashion to reduce operation complexity during the intervention. Implantation is executed in a top-down and pullback fashion with good tactile feedback once the arms are deployed in the optimal position. Full recoverability and perfusion is maintained throughout the entire time.

### Procedures

**Figure 2:**
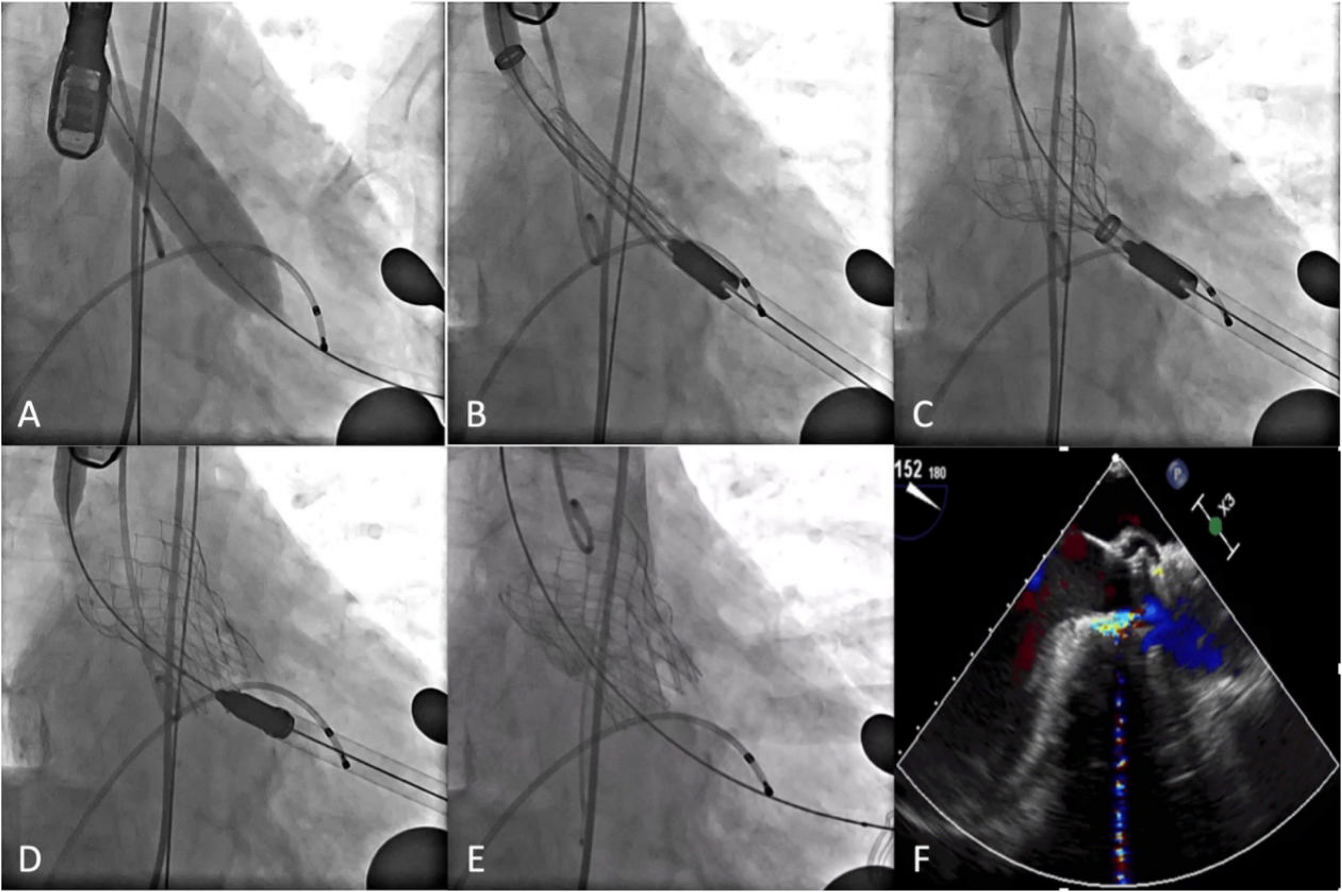
Procedural details. **(A)** Pre-dilation. **(B)** Xcor system was positioned. **(C)** The supporting arms were released. **(D)** The prosthesis was fully unfolded. **(E,F)** DSA and transesophageal echocardiography showed the position and morphology of the prosthesis.

The procedures were performed in a hybrid OR under general intravenous anesthesia and transesophageal echocardiography (TEE). The left femoral artery was punctured, and a pigtail catheter was inserted into the aortic root. The apex of the heart was located by x-ray examination. The chest was opened by a 3 cm incision through the left lateral 4th intercostal space. The pericardium was incised and suspended, the apex of the heart was pre-stitched with two deep U-stitches armed with Teflon felts, the apex of the heart was punctured and a 2.6 m straight head guide wire and a multifunctional catheter was inserted into the left ventricle. The 2.6 m straight head guide wire was then passed through the aortic valve and the multifunctional catheter was advanced into the descending aorta. The guide wire was then replaced by a Lunderquist or Superstiff guide wire and the multifunctional catheter removed. If necessary, the annulus was pre-dilated using a suitable balloon under a short period of rapid pacing (Figure 2A). The Xcor delivery system was then inserted and the Xcor valve system was released under fluoroscopic guidance (Figures 2B–D). After the Xcor valve system was implanted, the implant position and valve function were evaluated using fluoroscopy and TEE (Figures 2E,F). If there was ≥ mild paravalvular leakage (PVL) or mean PG ≥ 20 mmHg, post-dilation was used to improve the result. Additionally, more procedural details could be seen in the Supplementary Video S1.

### Endpoints

Valve Academic Research Consortium -3 (VARC-3) defined combined endpoints were reported (8): 30 days device success (technical success, freedom from mortality, freedom from reintervention related to the device, hemodynamic device performance (mean gradient <20 mmHg, peak velocity <3 m/s). Procedural success was defined as the correct positioning of a single Xcor valve in the annulus and successful retrieval of the delivery system from the patient. In addition, procedural success was defined as no death, stroke, myocardial infarction, or acute kidney failure within 72 h after the procedures or before discharge. Major vascular complications were reported according to VARC-3 criteria at hospital discharge (8). Need for permanent pacemaker implantation (PPI) and stroke (VARC-3 all stroke) were reported at 30 days. Functional status was evaluated using the BNP level and NYHA Class. Quality of life was assessed using the Short Form-12 (SF-12) Health Survey score.

### Data collection

Demographics, procedural details, intra-hospital course and adverse events were prospectively recorded according to the VARC-3 recommendations in a dedicated database (8). Data were evaluated by an independent Clinical Events Committees (CEC) consisting of three experts who were not involved in clinical trials, whose adjudication provides a standard for the systematic and unbiased assessment of endpoints. The premarket study was registered in China under the registration number: ChiCTR2200065593.

Echocardiography examination at discharge and at 30-day follow-up was performed with two dimensional and doppler transthoracic echocardiography (TTE) in accordance with the imaging recommendations of prosthetic heart valves (9). The EOA was measured at discharge by TTE using the continuity equation.

### Statistical analysis

All statistical analyses were performed using SPSS version 26.0 (Armonk, USA). Continuous variables were expressed as mean ± standard deviation, and the paired Student *t* test was used for comparison between groups. Categorical variables were expressed as percentage or frequency, and the Wilcoxon test was used for comparison between groups. Bilateral *p* values < 0.05 were considered statistically significant.

## Results

### Baseline characteristics

130 consecutive patients from 11 institutions were included in the study. Table 1 lists the baseline characteristics. The average age was 71.2 ± 4.4 years, 55.4% of patients were male, and the Society of Thoracic Surgeons (STS) score was 8.0 ± 3.9%. Of the overall cohort, 88.4% of patients were in NYHA Class III or IV, N-terminal pro-B type natriuretic peptide (NT-proBNP) was 3,003.2 ± 5,439.5 ng/L. A previous history of coronary artery disease, cerebrovascular disease, and chronic obstructive pulmonary disease was present in 43.1%, 14.6% and 43.1% of the patients, respectively. 13.8% of the patients presented with atrial fibrillation, and 3.1% with a permanent pacemaker. Baseline characteristics of bicuspid AV (BAV) patients were not significantly different from those of TAV patients except for previous history of percutaneous coronary intervention, cerebrovascular disease, and chronic obstructive pulmonary disease (Supplementary Table S2).

**Table 1:** Baseline characteristics of patients underwent TAVR using Xcor system (*n* = 130).

Characteristics	All (*n* = 130)
Age, y	71.2 ± 4.4
Male, *n* (%)	72 (55.4)
Body mass index, kg/m^2^	22.9 ± 3.2
STS score, %	8.0 ± 3.9
Log-EuroSCORE	6.2 ± 2.4
NYHA class ≥ III	115 (88.4)
NT pro-BNP, ng/L	3,003.2 ± 5,439.5
Coronary artery disease, *n* (%)	56 (43.1)
Myocardial infarction, *n* (%)	1 (0.8)
Percutaneous coronary intervention, *n* (%)	7 (5.4)
Coronary artery bypass grafting, *n* (%)	1 (0.8)
Cerebrovascular disease, *n* (%)	19 (14.6)
Chronic obstructive pulmonary disease, *n* (%)	56 (43.1)
Atrial fibrillation, *n* (%)	18 (13.8)
Permanent pacemaker implantation, *n* (%)	4 (3.1)

TAVR, transcatheter aortic valve replacement; STS, Society of Thoracic Surgeons; EuroSCORE, European system for cardiac operative risk evaluation; NT pro-BNP, N-terminal pro-brain natriuretic peptide.

Baseline CTA and TTE parameters are listed in Table 2. The mean diameter and perimeter of the annulus was 24.7 ± 2.6 mm and 77.4 ± 7.9 mm, respectively. The mean diameter of the LVOT and the distance of the left- and right coronary artery from the annulus was 25.3 ± 3.2 mm, 13.6 ± 3.7 mm and 16.7 ± 3.3 mm, respectively. Notably, the percentage of BAV in all patients reached 58.5% and LVEF was 58.7 ± 12.0%. The peak velocity was 4.7 ± 0.8 m/s, and the mean PG was 55.9 ± 21.5 mmHg. In addition, the procedural imaging measurements in BAV patients were similar to those in TAV patients (Supplementary Table S3).

**Table 2:** Preprocedural imaging assessments of patients underwent TAVR using the xcor system (*n* = 130).

Characteristics	All (*n* = 130)
Computed tomography angiography measurements	
Annular diameter, mm	24.7 ± 2.6
Annular perimeter, mm	77.4 ± 7.9
Left ventricular outflow tract diameter, mm	25.3 ± 3.2
Left coronary artery height, mm	13.6 ± 3.7
Right coronary artery height, mm	16.7 ± 3.3
Transthoracic echocardiography measurements	
Bicuspid aortic valve, %	76 (58.5)
Peak velocity of aortic valve, m/s	4.7 ± 0.8
Maximum pressure gradient, mmHg	90.2 ± 32.7
Mean pressure gradient, mmHg	55.9 ± 21.5
Effective orifice area, cm^2^	0.7 ± 0.2
Left ventricular ejection fraction, %	58.7 ± 12.0

TAVR, transcatheter aortic valve replacement.

### Procedural details and Xcor function

The procedural characteristics are shown in Table 3. All patients underwent TAVR under general anesthesia without cardiopulmonary bypass, and the mean device time (from apex puncture to apex closure) was 7.0 ± 2.3 min. The proportion of 23 mm, 26 mm and 29 mm Xcor used during the procedures was 37.7% (*n* = 49), 36.2% (*n* = 47) and 26.2% (*n* = 34), respectively. Device success was achieved in 128 of the 130 patients (98.5%). In two patients the implanted THV did not achieve the desired outcome (mean PG < 20 mmHg or peak velocity <3 m/s). The procedural success was 97.7% (*n* = 127), two patients died before discharge (1 from acute heart failure, and 1 from multiple organ dysfunction), and the third patient with preprocedural renal insufficiency developed acute kidney failure. None of the patients experienced intraprocedural malposition, or annular rupture or needed intraprocedural conversion to surgery, or a second THV implantation. Postprocedural balloon dilatations were performed in more than 80% of the patients. Of the 130 patients with implanted devices, 4.6% (*n* = 6) developed mild PVL, and 3.0% (*n* = 4) developed moderate PVL. BAV patients achieved similar procedural outcomes as TAV patients (device success rate: 98.7% vs. 98.2%, *P* = 0.929; procedural success rate: 97.4% vs. 98.2%, *P* = 0.816).

**Table 3:** Procedural characteristics of patients underwent TAVR using the xcor system (*n* = 130).

Characteristics, *n* (%)	All (*n* = 130)
Prosthesis size 23 mm	49 (37.7)
Prosthesis size 26 mm	47 (36.2)
Prosthesis size 29 mm	34 (26.2)
Device success	128 (98.5)
Procedural success	127 (97.7)
Device time, min	7.0 ± 2.3
Conversion to surgery	0 (0)
Mal-positioning	0 (0)
Annular rupture	0 (0)
Device embolization	0 (0)
Valve-in-valve implantation	0 (0)
PVL ≥ mild intra-procedural	10 (7.7)

TAVR, transcatheter aortic valve replacement; PVL, paravalvular leakage.

### Clinical outcomes

**Figure 3:**
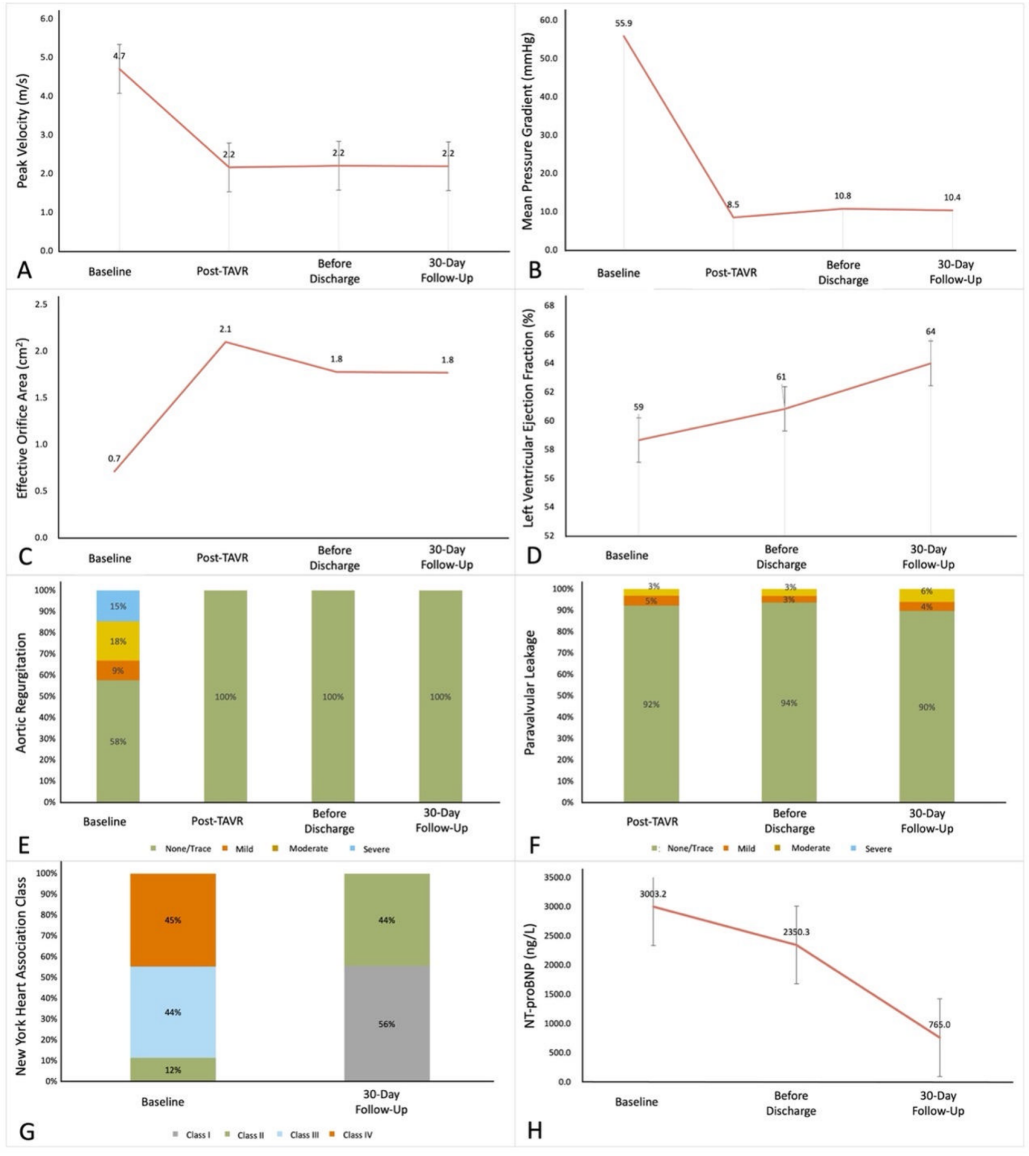
The improvements of 30-day follows-up compared to pre-TAVR results. **(A)** Aortic valve peak velocity. **(B)** Mean pressure gradient. **(C)** Effective orifice area. **(D)** Left ventricular ejection fraction. **(E)** Aortic regurgitation. **(F)** Paravalvular leakage. **(G)** New York Heart Association class. **(H)** N-terminal pro-brain natriuretic peptide. *P*-value determined from the paired Student *t*-test or the Wilcoxon signed rank test.

The outcome during the in-hospital stays (7.5 ± 3.7 days) and during follow-up are shown in Table 4 and Figure 3. Before discharge, the all-cause mortality was 1.5%, no patients exhibited myocardial infarction, stroke, or life-threatening bleeding.

**Table 4:** Clinical outcomes at 30-day follow-up of patients underwent TAVR using xcor system (*n* = 125).

Items, *n* (%)	All (*n* = 130)
All-cause mortality	5 (3.8)
Major adverse cardiovascular events	6 (4.6)
Myocardial infarction	0 (0)
Stroke	0 (0)
Life-threatening bleeding	0 (0)
Major vascular complications	1 (0.8)
Acute kidney injury	0 (0)
New permanent pacemaker implantation	1 (0.8)

Major adverse cardiovascular events include myocardial infarction, III atrioventricular conduction block, sinus arrest, acute coronary syndrome, atrial/ventricular fibrillation, and reinterventions.

At 30-day follow-up, all-cause mortality was 3.8%, 3 patients died of COVID-19, 1 case of acute heart failure, and 1 case of multiple organ dysfunction. Major adverse cardiovascular events occurred in 6 patients (4.6%), including 2 cases of atrial fibrillation, 1 case of acute heart failure, 1 case of sinus arrest, 1 case of acute coronary artery syndrome, and 1 case of ventricular fibrillation. One patient (0.8%) showed a major vascular complication, and one patient (0.8%) needed a permanent pacemaker implantation because of preprocedural sick-sinus syndrome. At 30 days follow up the peak velocity had decreased from 4.7 ± 0.8 m/s to 2.2 ± 0.4 m/s (*P* < 0.001 and the mean PG from 55.9 ± 21.5 mmHg to 10.4 ± 4.4 mmHg (*P* < 0.001). Accordingly, the effective orifice area (EOA) increased from 0.7 ± 0.2 cm^2^ to 1.8 ± 0.4 cm^2^ (*P* < 0.001). Furthermore, all surviving 125 patients were in NYHA Class ≤ II. All hemodynamic data are depicted in Supplementary Table S4 prior to the procedure, at discharge and at 30 day follow up.

At 30 days the following parameters showed no significant difference comparing BAV to TAV results: mean peak velocity (2.2 ± 0.4 m/s vs. 2.1 ± 0.4 m/s, *P* = 0.630), pressure gradient (10.8 ± 4.5 mmHg vs. 10.4 ± 4.4 mmHg, *P* = 0.648), EOA (1.7 ± 0.4 cm^2^ vs. 1.9 ± 0.5 cm^2^, *P* = 0.061), LVEF (64.6 ± 8.6% vs. 63.2 ± 9.0%, *P* = 0.382) and NT-proBNP (557.0 ± 589.4 ng/L vs. 771.6 ± 1,055.9 ng/L, *P* = 0.123), the proportion of  ≥mild central regurgitation (11.8% vs. 13.1%, *P* = 0.763),  ≥mild PVL (9.2% vs. 9.2%, *P* = 0.984) and NYHA Class ≤Ⅱ (100.0% vs. 100.0%, *P* = 1.000) had no in BAV patients.

## Discussion

Since the first TAVR in 2002 (10), the procedure has been recognized as the preferred treatment for patients with AS who are at high or prohibitive risk for SAVR (1–5, 11, 12). It has been demonstrated that compared with SAVR, TAVR results in lower rates of major bleeding, acute kidney injury, and new onset atrial fibrillation but in higher rates of conduction abnormalities, paravalvular leak, and vascular complications (13–20).

Currently, most existing THV systems rely solely on radial force to hold the implanted THV in place of the native AV. However, since the native AV is not removed and most of the patients show asymmetric calcifications, the device may be squeezed by the surrounding anatomical structures after implantation, so that the position and axiality of THV may not meet the expectations (6, 7). Inadequate anchoring may lead to an increased risk of THV displacement, while oversizing or post-dilation may result in annular rupture. Importantly, the above factors may eventually lead to embolism, increase the need of a new permanent pacemaker implantation (PPI) and reduced THV durability, thus affecting the long-term prognosis (21).

The Xcor system has the unique design feature of supporting arms. The supporting arms control the height of implantation so that a deep positing of the Xcor device is extremely unlikely to occur. Procedural factors like implant depth and procedural manipulations, such as re-sheathing have been shown to interfere with the conduction system and can result in the subsequent need for PPI. In the present study PPI was 0.8%, which is lower than observed after implantation of any other TAV device. Only one patient suffering from preexisting sick sinus syndrome needed PPI. Reported PPI rates for the Evolut PRO (11.8%), ACURATE NEO2 (15.0%), and SAPIEN 3 (13.3%) in prospective studies with a similar high- or extreme-risk population are considerable higher (22–24).

The Xcor Transcapical System features a self-expanding nitinol stent designed in a monolithic configuration, featuring six expandable arms symmetrically positioned within the central portion of the stent. As of the study period, the system is available in three distinct sizes (23, 26, and 29, measured as the maximum diameter of the inflow), effectively covering an annular range spanning from 19–28 mm.

A fundamental characteristic of the Xcor stents lies in its expandable arms, which lack any undercuts, thus ensuring complete recoverability. These arms play a pivotal role in facilitating precise positioning, self-alignment, and secure anchoring of the prosthesis. Employing a form-fitting principle, the arms serve to diminish the necessary radial force within the conical inflow of the stent. Furthermore, by ensuring minimal protrusion into the left ventricular outflow tract (LVOT), they alleviate any undue pressure on the left bundle branch (LBB).

The expandable arms, assure a supra-annular valve function with three bovine pericardial leaflets. Their design enables, in conjunction with large diamante cells allow for easy coronary access. Central co-axial self-alignment, along with the inclusion of a Dacron outer-skirt measuring a total height of 10–11 mm, bestows an excellent sealing capability, effectively averting paravalvular leakage. It is noteworthy that all valve sizes are compatible with the same 23 French delivery system, designed with simplicity and efficiency in mind to streamline the operative procedure. The implantation process follows a top-down and pullback technique, offering tactile feedback upon the arms' optimal deployment. Full recoverability and uninterrupted perfusion are maintained throughout the entire duration of the intervention.

Device success with the Xcor system was achieved in 98.5%, (*n* = 128) and procedural success in 97.7% (*n* = 127). None of the patients experienced intraprocedural mal-positioning, re-sheathing, annular rupture, intraprocedural conversion to surgery, or a second THV implantation. The hemodynamic parameters improved significantly, showing a large EOA and low gradients at 30 days follow-up. The design of the supporting arms improves procedural success rate and optimizes co-axiality to a certain extent, improving EOA and mean PG. Of the 130 patients with implanted devices, 4.6% (*n* = 6) developed mild PVL, and 3.0% (*n* = 4) developed moderate PVL. The incidence of PVL after TAVR is 12%–30%, which is significantly higher than that of SAVR (25). More than moderate PVL due to poorer THV fitting is a major risk factor for poor prognosis (25). In the Xcor system, the six supporting arms optimize radial support while achieving accurate implantation to reduce the incidence of THV displacement. Notably, BAV patients achieved similar procedural outcomes as TAV patients (device success rate: 98.7% vs. 98.2%, *P* = 0.929; procedural success rate: 97.4% vs. 98.2%, *P* = 0.816). Studies have shown that the proportion of AS patients with BAV morphology in China is close to 50% (26). BAV is characterized by heavy calcification burden, large annuli and tortuous deformity of the aorta, which makes the procedures more difficult than that of TAV patients (27). The Xcor system forms fit between the supporting arms and the surrounding anatomy of the native AV during release to achieve longitudinal centralization and self-centered effect, and is suitable for both TAV and BAV patients.

In this study, the STS score was 8.0 ± 3.9%, but the all-cause mortality at 30-day follow-up was only 3.8% with no stroke, which was better than the outcomes of other large clinical studies (21–24). An all-cause mortality rate of 3.8% at 30 days in this cohort compares favorably with other transcatheter valves (1.7%–3.3%) studied in a similar patient population (21–24).

Event rates were comparable with other published outcomes of the SAPIEN 3, Evolut PRO, and ACURATE neo2 valves, including stroke (0.9%–1.7%), life-threatening bleeding (5.0%–11.7%), and major vascular complications (3.3%–10.0%), for a high or extreme surgical risk population and suggests that the novel Xcor device performs similarly to other contemporary THVs.

## Study limitations

The follow-up time of this study is relatively short, and the measurements of hemodynamic parameters lacks the support of a unified core laboratory, requiring further studies to determine the safety and reliability of its application in AS. Importantly, we used the transapical approach mainly because the new bioprosthetic valve design led us to believe that the transapical approach might increase the success rate of the surgery to a certain extent while reducing the learning curve for surgeons. Meanwhile, we are actively developing the second-generation product for the transfemoral approach, striving to ensure the mainstream approach while further improving the clinical performance of the valve.

## Conclusions

Overall, the 30-day follow-up in this premarket, prospective, multicenter study showed that the procedural and short-term clinical outcomes with the Xcor system were promising, with low all-cause mortality and low major adverse cardiovascular events. Clinical outcomes for BAV and TAV patients were not different. These results suggest the feasibility of the system in high-risk patients with severe AS. The PPI with the Xcor device is at an extraordinary low 0.8% which has not yet been achieved with any other TAV device.

## Data Availability

The original contributions presented in the study are included in the article/supplementary material, further inquiries can be directed to the corresponding author.
