# Supplementary material for: Early experience with a novel transapical transcatheter aortic valve system in patients with severe aortic stenosis: a prospective, multicenter study
Source: Front Cardiovasc Med. 2025 Mar 21;12:1457180. doi: 10.3389/fcvm.2025.1457180 (PMC11968724; doi:10.3389/fcvm.2025.1457180)
Supplement: Supplementary file 1 [file Datasheet1.pdf]

Supplementary Materials

Supplementary Table 1 Inclusion and exclusion criteria

Inclusion criteria	1) Age ≥ 70 years old;
	2) Severe aortic stenosis: Mean pressure gradient ≥ 40 mmHg (1 mmHg = 0.133kPa) or aortic annular area < 1.0 cm^2;
	3) New York Heart Association Class ≥ II;
	4) High risk for surgical aortic valve replacement.
Exclusion criteria	1) Acute myocardial infarction occurred within 1 month;
	2) Aortic root anatomy and pathological changes are not suitable for bioprosthetic valve implantation;
	3) Any therapeutic traumatic heart surgery within 1 month;
	4) Ascending aortic aneurysm diameter ≥ 50 mm;
	5) Coagulation dysfunction;
	6) Hemodynamic instability;
	7) Hypertrophic cardiomyopathy;
	8) Left ventricular ejection fraction < 20%;
	9) Echocardiography indicated the presence of intracardiac mass, thrombus, or neoplasm;
	10) Active peptic ulcer or history of upper gastrointestinal bleeding within 3 months;
	11) Cerebrovascular accident occurred within 3 months;
	12) Infectious endocarditis;
	13) The expected survival time is less than 1 year.

Supplementary Table 2 Baseline characteristics of tricuspid versus bicuspid aortic valve patients (n = 130)

Characteristics	TAV (n = 54)	BAV (n = 76)	*P*-Value
Age, y	71.6	70.7	0.402
Male, n (%)	30（55.5）	42（55.3）	0.942
Body mass index, kg/m^2	22.9 ± 3.1	22.9 ± 3.3	0.851
STS score, %	8.0 ± 3.6	8.0 ± 4.2	0.961
Log-EuroSCORE	6.2 ± 2.4	6.4 ± 2.3	0.763
NYHA class ≥ III	48 (88.8)	67 (88.2)	0.833
NT pro-BNP, ng/L	3003.2±5439.5	2656.1±4936.5	0.633
Coronary artery disease, n (%)	20（37.0）	36（47.4）	0.238
Myocardial infarction, n (%)	0 (0)	1（1.3）	1.000
Percutaneous coronary intervention, n (%)	6（11.1）	1（1.3）	**< 0.001**
Coronary artery bypass grafting, n (%)	1（1.9）	0（0）	1.000
Cerebrovascular disease, n (%)	0 (0)	19（14.6）	**< 0.001**
Chronic obstructive pulmonary disease, n (%)	14（25.9）	42（55.3）	**< 0.001**
Atrial fibrillation, n (%)	8（14.8）	10（13.2）	0.714
Permanent pacemaker implantation, n (%)	4（7.4）	0（0）	0.082

TAVR, transcatheter aortic valve replacement; STS, Society of Thoracic Surgeons; EuroSCORE, European system for cardiac operative risk evaluation; NT pro-BNP, N-terminal pro-brain natriuretic peptide; TAV, tricuspid aortic valve; BAV, bicuspid aortic valve.

Supplementary Table 3 Preprocedural imaging assessments of tricuspid versus bicuspid aortic valve patients (n = 130)

Characteristics	TAV (n = 54)	BAV (n = 76)	*P*-Value
**Computed tomography angiography measurements**			
Annular diameter, mm	24.7 ± 2.6	24.7±2.6	0.980
Annular perimeter, mm	77.4 ± 7.9	77.4±7.8	0.954
Left ventricular outflow tract diameter, mm	25.3 ± 3.2	25.4±3.3	0.893
Left coronary artery height, mm	13.6 ± 3.7	14.3±4.1	0.195
Right coronary artery height, mm	16.7 ± 3.3	16.6±3.6	0.810
**Transthoracic echocardiography measurements**			
Peak velocity of aortic valve, m/s	4.7 ± 0.8	4.9 ± 0.9	0.229
Maximum pressure gradient, mmHg	90.2 ± 32.7	96.1 ± 36.9	0.234
Mean pressure gradient, mmHg	55.9 ± 21.5	59.8 ± 23.8	0.246
Effective orifice area, cm^2	0.7 ± 0.2	0.7 ± 0.2	0.649
Left ventricular ejection fraction, %	58.7 ± 12.0	58.6 ± 12.2	0.983

TAVR, transcatheter aortic valve replacement.

Supplementary Table 4 Echocardiographic Outcomes at Post-TAVR, Discharge and 30 Days (n = 130)

Characteristics	Post-TAVR				Before Discharge			
	TAV (n = 54)	BAV (n = 76)	Total （n=130）	*P*-Value	TAV (n = 54)	BAV (n = 76)	Total （n=130）	*P*-Valu
Peak velocity of aortic valve, m/s	2.2 ± 1.5	2.1 ± 1.1	2.2 ± 1.3	0.884	2.2 ± 0.5	2.2 ± 0.5	2.2 ± 0.5	0.700
Maximum pressure gradient, mmHg	16.4 ± 8.0	16.0 ± 8.3	16.2 ± 8.2	0.946	19.7 ± 9.6	20.8 ± 9.1	20.3 ± 9.2	0.827
Mean pressure gradient, mmHg	9.0 ± 4.3	8.2 ±4.5	8.5 ±4.4	0.660	10.6 ±5.5	11.0 ±5.2	10.8 ±5.3	0.927
Effective orifice area, cm^2	2.0 ± 0.6	2.2 ± 0.8	2.1 ± 0.8	0.417	1.7 ± 0.4	1.8 ± 0.5	1.8 ± 0.5	0.554
Left ventricular ejection fraction, %	-	-	-	-	59.6 ± 11.7	61.7 ± 9.6	60.8 ± 10.4	0.530

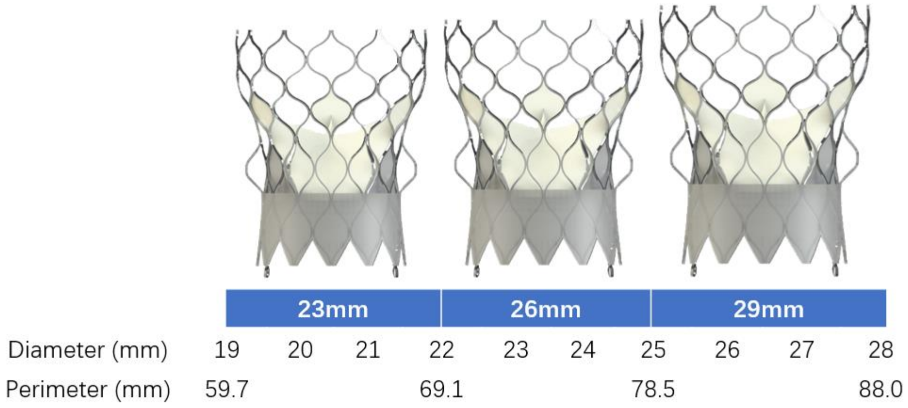

Supplementary Figure 1 The sizing chart of Xcor system (Saint Medical Technology Co., LTD., Nanjing, China) prothesis.

Supplementary Video 1 Procedural details.
